# Does improving maternal knowledge of vaccines impact infant immunization rates? A community-based randomized-controlled trial in Karachi, Pakistan

**DOI:** 10.1186/1471-2458-11-239

**Published:** 2011-04-17

**Authors:** Aatekah Owais, Beenish Hanif, Amna R Siddiqui, Ajmal Agha, Anita KM Zaidi

**Affiliations:** 1 Department of Paediatrics and Child Health, Aga Khan University, Karachi, Pakistan; 2 Department of Community Health Sciences, Aga Khan University, Karachi, Pakistan

## Abstract

**Background:**

In Pakistan, only 59-73% of children 12-23 months of age are fully immunized. This randomized, controlled trial was conducted to assess the impact of a low-literacy immunization promotion educational intervention for mothers living in low-income communities of Karachi on infant immunization completion rates.

**Methods:**

Three hundred and sixty-six mother-infant pairs, with infants aged **≤ **6 weeks, were enrolled and randomized into either the intervention or control arm between August - November 2008. The intervention, administered by trained community health workers, consisted of three targeted pictorial messages regarding vaccines. The control group received general health promotion messages based on Pakistan's Lady Health Worker program curriculum. Assessment of DPT/Hepatitis B vaccine completion (3 doses) was conducted 4-months after enrollment. A Poisson regression model was used to estimate effect of the intervention. The multivariable Poisson regression model included maternal education, paternal occupation, ownership of home, cooking fuel used at home, place of residence, the child's immunization status at enrollment, and mother's perception about the impact of immunization on child's health.

**Results:**

Baseline characteristics among the two groups were similar. At 4 month assessment, among 179 mother-infant pairs in the intervention group, 129 (72.1%) had received all 3 doses of DPT/Hepatitis B vaccine, whereas in the control group 92/178 (51.7%) had received all 3 doses. Multivariable analysis revealed a significant improvement of 39% (adjusted RR = 1.39; 95% CI: 1.06-1.81) in DPT-3/Hepatitis B completion rates in the intervention group.

**Conclusion:**

A simple educational intervention designed for low-literate populations, improved DPT-3/Hepatitis B vaccine completion rates by 39%. These findings have important implications for improving routine immunization rates in Pakistan.

## Background

Vaccinating infants against childhood communicable diseases is one of the most cost-effective public health interventions worldwide [1]. Pakistan's Expanded Program on Immunization (EPI) schedule involves administrating BCG/OPV at birth, three doses of DPT/OPV/Hepatitis B vaccines at 6, 10 and 14 weeks of age, and measles vaccine at 9 and 15 months of age. In Pakistan, children receive vaccinations through EPI at fixed primary health centers. Additional supplementary activities are undertaken through outreach efforts, such as National Immunization Days. Recently, Pakistan has added the *Haemophilus influenzae *type b vaccine to be administered with the three doses of DPT/Hepatitis B vaccines. Despite recent efforts, immunization coverage rates in Pakistan remain low, with 59-73% of children aged 12 - 23 months receiving all three doses of DPT/Hepatitis B vaccine [2,3]. Therefore, low-cost innovative interventions, which can be implemented within Pakistan's existing health care infrastructure, are needed.

Many studies have looked at factors that affect immunization completion rates. Low parental, specifically maternal literacy and knowledge regarding vaccines and immunization schedule, poor socioeconomic status, and residence in rural areas are associated with low immunization coverage [4-12]. Health provider factors that have been associated with increased immunization drop-out rates include parental difficulty of access to healthcare services and inadequate supervision of healthcare staff at health facilities [4-6,8]. Retention of proof of immunization by the infants' families has been associated with improved immunization coverage and facilitates documentation of vaccination status [6,10,11].

Increasing health awareness, knowledge about diseases, and their prevention or management has successfully improved many different health outcomes in high-income countries, especially among less literate populations [13-16]. Educational interventions promoting vaccine use have also proven cost-effective in improving immunization coverage rates in these settings [17,18]. However, there is limited data available from low-income countries [12]. Usman et al [12] recently reported findings from a randomized controlled trial in urban Pakistan evaluating the effect of center-based education to mothers of infants presenting at primary healthcare centers for first dose of DPT vaccine. The 2-3 minutes education session, conducted by trained study staff, emphasized the importance of completing the immunization schedule, and improved immunization completion rates by 31% among the cohort of infants whose mothers presented to an immunization center for their first vaccine [12].

To our knowledge, no study has looked at the impact of home-based vaccine promotion education among a population of mothers of newborns at high risk for not seeking immunization services for their children. Our study aims to close this gap. The main objective of this study was to assess the effect of short, home-based information sessions on importance of vaccines on DPT-3/Hepatitis B immunization rates in low-income urban and peri-urban communities in Karachi, Pakistan known for very low demand and care-seeking for vaccination services.

## Methods

### Study setting

This was a multi-site community-based, randomized controlled educational intervention trial conducted at five low-income sites in Karachi. Among these, one community was urban, whereas the other four were peri-urban, located about 45 minutes travel outside of Karachi. The population in the study areas has low literacy, with only 24% of the population being literate. The total combined population of all five study sites is approximately 260,000, with high infant and maternal mortality rates. The major income generating activities include fishing and livestock rearing, or employment in local small industries (garment and leather).

The Department of Pediatrics and Child Health, Aga Khan University has well-established household-based surveillance for pregnancy and neonatal outcomes in these areas. A demographic surveillance round of the entire area is completed every three months. All pregnant women identified during this surveillance are visited frequently around the time of birth so that new births can be captured.

### Eligibility and enrollment of participants

All mothers living in the study areas, and having a live child ≤ 6 weeks old, were eligible to be enrolled in the study. Twin births, infants > 6 weeks of age, or infants born to mothers living outside the study surveillance areas were excluded. The cutoff of 6 weeks was used to ensure that the intervention was implemented before the first dose of DPT/Hepatitis B came due.

Mothers of possibly eligible infants were identified through computerized surveillance databases of pregnant women and newborns maintained at each site. Families were approached with the help of local women, trained as community health workers (CHWs). The CHWs also obtained verbal consent from the mother of each eligible infant. The study protocol was approved by the Ethical Review Committee of the Aga Khan University. Informed consent was obtained from each participant at enrollment, and no breaches of confidentiality occurred.

Each mother-infant pair, who consented to participate in the study, was assigned a unique study identification number. Information on household demographics and socio-economic status was collected using a pre-tested structured questionnaire. Data collected on the infant included age, sex, place of birth, and health status. The baseline interview also recorded mother's knowledge and beliefs about vaccines. A follow-up questionnaire was used to assess the outcome. Subjects were enrolled from August 2008 to November 2008. Study participants were followed up for assessment of outcome from December 2008 to March 2009, with each individual mother-infant pair approached four months after the educational intervention session.

### Randomization

Randomization lists, stratified for each of the five enrollment sites were generated by a computer and provided to the CHWs Upon consent, mother-infant pairs were assigned either to intervention or control arms through block randomization (n = 4), according to the computer-generated list. As the intervention was educational, blinding of study staff and participants was not possible. Outcome assessment was done by an investigator (BH) at each participant's house, four months after initial enrollment. The investigator was blinded to the exposure status of participants.

### Intervention

To address the needs of low literacy populations, easy-to-understand pictorial cards, using very simple language, to convey three key messages as part of the educational intervention were designed. The first key message highlighted how vaccines save children's lives. The second message provided logistic information about the address and location of the local vaccination centers. The third key message emphasized the significance of retaining immunization cards, and the role they could play at the time of the child's school admissions. A copy of these pictorial messages was left with the mother. These messages took about 5 minutes to impart, and were given by the trained CHWs to each participant at their household.

The control group verbally received the general health promotion messages adapted from the curricula developed by the Pakistan Ministry of Health for the Lady Health Worker Program. These messages included information on hand-washing, breast-feeding, clean water, benefits of using oral rehydration solutions during diarrhea, bringing the infant to nearby health center when there are symptoms of acute respiratory illnesses, importance of antenatal check-ups for mothers, and some general information on vaccines. These messages were also given by trained CHWs. The length of each educational session in the control group was approximately 10-15 minutes.

### Study outcome

The study outcome in each study group was the immunization status of DPT-3/Hepatitis B at 4 months after enrollment (4 to 5 months of infant's age). Immunization rates of DPT-3/Hepatitis B vaccines for intervention and control groups were assessed by an investigator, and were divided into two categories:

1) Infants receiving all three doses of DPT/Hepatitis B vaccines (assessed through vaccination cards) were considered "DPT-3/Hepatitis B fully immunized".

2) Infants missing any dose of DPT/Hepatitis B or who had lost their vaccination cards were termed"DPT-3/Hepatitis B non-immunized".

A participant was considered to be "DPT-3/Hepatitis B fully immunized" only if the mother/caretaker of the child was able to produce an EPI-issued or another health facility-issued vaccination card. Verbal responses of mothers regarding vaccine receipt without documentation on a vaccination card were not considered satisfactory evidence of their infant being fully immunized.

### Sample size

We assumed a DPT-3/Hepatitis B immunization rate of 55% in the control group, and hypothesized a difference of 15% in the immunization rates between the intervention and control group. With 80% power and α = 0.05, we estimated a sample size of 163 in each arm. Adjusting for possible lost to follow-up, we enrolled 183 mother-infants pairs in each study group.

### Statistical analysis

Statistical analysis was performed using SAS Version 9.2 (SAS Institute, Inc., Cary, NC). Baseline characteristics of study participants were compared using proportions. Unadjusted risk ratio (RR) and 95% confidence interval (CI) were estimated for the study outcome (DPT-3/Hepatitis B fully immunized) using bivariate Poisson regression [19,20]. A multivariable Poisson regression model was built to assess the association between the study outcome, the study group and all other variables which were considered to be significantly associated with the study outcome at the bivariate level (p ≤ 0.20). All variables, having bivariate association with the study outcome (p ≤ 0.20) were also tested for interaction. The final model was interpreted using adjusted RR and corresponding 95% CI. The number needed to treat (NNT) in order to increase the completion of DPT-3/Hepatitis B immunization by one child was also estimated [21,22].

## Results

**Figure 1:**
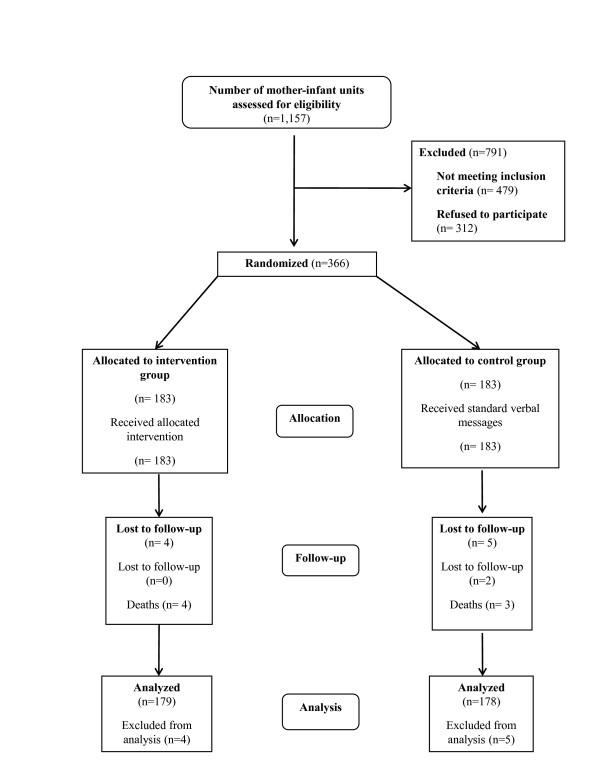
**Flow diagram of study participant screening, allocation and follow-up**.

A total of 1157 mother-infant pairs were identified from surveillance databases at the five community sites were approached, and assessed for eligibility. Among these, 479 (41.4%) did not meet inclusion criteria, whereas 312 (27%) declined participation in the study (Figure 1), resulting in 366 (183 in each study arm) children being available for randomization. Four infants were lost to follow-up from the intervention group, and five were lost to follow-up from the control group during the study period and were excluded from the analysis. Therefore, 179 enrolled infants were included in the analysis from the intervention group and 178 from the control group (Figure 1). The distribution of enrolled mother-infant pairs among the five study sites was weighted to represent population size in each area and was as follows: Community A = 103; Community B = 96; Community C = 71; Community D = 47; and Community E = 40.

The distribution of baseline characteristics of the participants in the intervention and control arms is summarized in Table 1. No significant differences were observed between the two groups, although the proportion of mothers who had received no formal education was higher in the control group compared to those in the intervention group (75% vs. 66%). History of receipt of BCG vaccine and OPV (first dose) at birth was similar in both groups (76.4% in the control group vs. 77.1% in the intervention group). After the 4-month follow-up period, 129 (72.1%) infants in the intervention group had completed primary immunization with three doses of DPT and Hepatitis B vaccines compared to 92 (51.7%) in the control group. Therefore, DPT-3/Hepatitis B immunization rates in the intervention group were improved by 39% (unadjusted RR = 1.39; 95% CI: 1.07 - 1.82) (Table 2). Vaccination cards were retained by 81% of the study participants in the intervention group, and by 69.1% of the participants in the control group.

**Table 1:** Distribution of baseline characteristics of study participants

	Control Group	Intervention Group	
	
	(*n *= 183)	(*n *= 183)	p-value
Age at enrollment (mean days)	22.4	25.7	0.01
Male child (%)	50.3	44.8	0.30
Child was immunized at enrollment	76.4	77.1	0.88
Age of mother (mean years)	25.1	25.9	0.17
Education of mother			0.07
No formal education (%)	74.9	66.1	
Primary (%)	12.0	20.8	
Middle and above (%)	13.1	13.1	
Occupation of mother			0.56
Housewife (%)	98.9	99.5	
Other professions (%)	1.1	0.5	
Education of father			0.09
No formal education (%)	54.6	53.6	
Primary (%)	20.8	13.7	
Middle and above (%)	24.6	32.8	
Occupation of father			0.54
Fishermen (%)	33.9	27.9	
Laborer (%)	20.2	22.4	
Businessman (%)	13.7	10.9	
Private job (%)	24.0	30.1	
Other profession (%)	8.2	8.7	
Ownership of house			0.80
Own (%)	79.2	80.3	
Rented (%)	20.8	19.7	
Construction material of house			0.79
Wooden structure or other (%)	6.6	5.5	
Tin (%)	21.3	25.1	
Concrete cement (%)	62.8	61.7	
Cement (%)	9.3	7.7	
Cooking fuel used in house			0.10
Wood or other (%)	20.8	14.2	
Natural gas (%)	79.2	85.8	
Place of birth			0.52
Home or other (%)	59.0	62.3	
Hospital (%)	41.0	37.7	
Mother knows about the impact of immunization on child's health			0.40
Don't know (%)	18.6	15.3	
It prevents serious illness (%)	81.4	84.7	
Mother knows where the nearest local immunization center is			0.34
No (%)	8.7	7.7	
Yes (%)	91.3	92.3	

**Table 2:** Effect of home-based vaccine education to mothers on study outcome

	Total N = 357	Received 3 doses of DPT/Hepatitis B vaccine		Unadjusted RR (95% CI)	Adjusted RR ^a ^ (95% CI)
				
		*n*	%		
Home-based vaccine education	179	129	72.1	1.39 (1.07-1.82)	1.39 (1.06-1.81)
General health promotion messages	178	92	51.7	1.00	1.00

^a ^Multivariable model adjusted for child's immunization status at enrollment.

In the multivariable model, the child's immunization status at enrollment was the only variable significantly associated with the study outcome (p < 0.05). Adjusting for this variable did not change the effect estimate (adjusted RR = 1.39; 95% CI: 1.06 - 1.81) (Table 2). Place of residence of the study participants (urban vs. semi-urban) was not associated with the study outcome (p = 0.08).

The number needed to treat (NNT) in order to increase the completion of DPT-3/Hepatitis B immunization by 1 child was also calculated. An estimated 5 mothers need to be educated in order to have one more child complete his/her DPT-3/Hepatitis B vaccinations.

We also assessed if our results were subject to misclassification bias due to the requirement of documentation of vaccine receipt through vaccination cards for the infant to be classified as fully immunized at outcome assessment, and those without vaccination card as not fully immunized. There were 10 cases (3 in the intervention group and 7 in the control group) that could have been misclassified as "DPT-3/Hepatitis B non-immunized" due to lack of a vaccination card, but whose mothers recalled receipt of all three doses. Including these 10 children in the "DPT-3/Hepatitis B fully immunized" group, 132 (73.7%) infants in the intervention group had completed primary immunization with three doses of DPT and Hepatitis B vaccine, compared to 99 (55.6%) in the control group. Therefore, DPT-3/Hepatitis B immunization rates in the intervention group were improved by 33% (unadjusted RR = 1.33; 95% CI: 1.02 - 1.72). In the multivariable model, the child's immunization status at enrollment was the only variable significantly associated with the study outcome (p < 0.05). After adjusting for it, DPT-3/Hepatitis B immunization rates in the intervention group were improved by 32% (adjusted RR = 1.32; 95% CI: 1.02 - 1.71).

## Discussion

This study demonstrates that providing vaccine-related targeted education to mothers at home is an effective and practical strategy to improve childhood immunization rates in low literacy settings such as ours. In this randomized controlled trial, a significant improvement in infant DPT-3/Hepatitis B vaccine immunization rates was observed in the group of mothers who received home-based education on the importance of vaccines, compared to those who received standard health promotion messages only.

In low-income countries, efforts to improve infant vaccination completion rates have focused primarily on supply and/or provider factors, with little focus on creating demand for infant immunization services. The major thrust of the supply-side interventions to improve vaccination rates has been through mass immunization campaigns. These campaigns have been successful in improving vaccine coverage rates [23]. However, there are certain drawbacks of mass campaigns such as those done for polio vaccine in Pakistan and other developing countries [24-26]. They lead to a misconception on the parents' part that the child will be delivered all vaccines at home [25], and can result in a decline in the number of visits to the immunization centers, paradoxically driving down routine immunization coverage rates[26]. Furthermore, these mass immunization campaigns have resulted in declining performance of routine EPI activities in Pakistan[27].

Educational interventions have been successful in raising awareness regarding vaccine and increasing demand. Jacobson et al [17] were successful in increasing pneumococcal vaccine coverage rates among the elderly, by using low-literacy pamphlets encouraging study participants to "ask your doctor about the pneumonia shot". Kimura et al [18] were able to increase influenza vaccine coverage among workers in long-term care facilities with the help of an educational campaign and provision of free vaccines. In Pakistan, Usman et al [12] reported an increase of 31% in DPT3 completion among infants of mothers who received primary healthcare center-based education on their first immunization visit.

The success of educational interventions in modifying health-seeking behavior may also be attributed to the focused nature of the interventions. This is certainly true in our study. The intervention group received a 5-minute educational session, focusing on the importance of immunization for a child's health. The control group, on the other hand, received a 10-15 minute verbal session on general child health promotion. Therefore, the group receiving the focused message may have been more likely to understand and retain its content, and modify their behavior, compared to the control group who may have had "information overload".

We observed that maternal knowledge/perception regarding importance of vaccines was significantly associated with higher DPT-3/Hepatitis B immunization rates (RR = 2.11; 95% CI: 1.33 - 3.34). Our results are consistent with findings of other studies [4,9-11]. Surprisingly, infants living in rented houses (considered a crude proxy for lower socioeconomic status) were more likely to have received all 3 doses of DPT-3/Hepatitis B vaccines as compared to infants living in houses owned by their families (RR = 1.26; 95% CI: 0.93 - 1.71). However, this association is not significant. Another explanation could be that many of the households in these populations are considered illegal squatters but claim ownership of the land on which they've built their house. Therefore, paradoxically, households in rental accommodation could actually be socio-economically better-off.

It is worth noting that even with the educational intervention specific to vaccines, only 72% of infants were fully immunized in the intervention group. This is because our study included very low-income, low-literacy populations of Sindh province where baseline immunization rates are 48% [2], much lower than the national reported figure of 73% [3]. The national average is a composite figure, including more prosperous and literate parts of the Pakistani population and the large province of Punjab which has a more functional EPI system and estimated vaccine coverage of 65% [2]. National surveys may overestimate or over-report vaccine coverage rates [28]. Different immunization centers use different vaccination cards, which differ in the design and method of recording proof of vaccination. This makes it difficult for data collectors to accurately determine an infant's immunization status. Furthermore, verbal reports, in lieu of vaccination cards, are often accepted as proof of immunization, but our experience using serological confirmation shows poor correlation between verbal recall and serological immunity [29]. Therefore, the true vaccine coverage rate for Pakistani children may be closer to the figure of 59% estimated by the recent Demographic and Health Survey of Pakistan [2]. Although ascertaining the reasons for low vaccine coverage was beyond the scope of this study, many barriers to improving immunization coverage remain in low-income communities and need to be systematically addressed.

Our study also has a few limitations. First, the third key message provided education on retaining vaccination cards. Therefore, mothers in the intervention group were 17% more likely to save the card and provide proof of vaccination for outcome assessment. However, as shown above, using the stringent proof-of-vaccination via card to determine outcome did not bias our results significantly if infants with maternal recall of vaccine receipt were also included as fully immunized. Including these infants as fully immunized, DPT-3/Hepatitis B immunization rates in the intervention group were still 18 percentage points higher than the control group.

A second limitation is the lack of blinding of CHWs and participants as the intervention was educational in nature. However, the investigator (BH) assessing the outcome four months after the intervention was administered was blinded to the exposure status of the study participants. Furthermore, chances of spillover effect, or contamination between the intervention and control arms were minimized by choosing mother-infant pairs from five different communities in Karachi, lowering the probability that households participating in our study with an eligible newborn would be located close to each other. We also observed a trend for mothers in the intervention group to be more educated compared to those in the control group (34% vs. 25%). However, this difference was not statistically significant and other measures of parental knowledge about vaccines did not favor the control group. Our study also had a high refusal rate (27%) which may have excluded participants less likely to accept vaccines from the trial. However, the most common stated reason for refusal was absence of the child's father when study staff visited the household for initial recruitment.

Our educational intervention has the potential to be cost-effective. The cost of the intervention per CHW was estimated to be Pakistan Rs. 80 ($1). This includes the cost of laminated colored pictorial cards used by the CHWs to educate the mothers in the intervention group, as well as pamphlets of the pictorial messages left at each participant's house. We also estimated the cost of scaling-up this intervention nationally, through the Lady Health Worker Program. Given that there are 100,000 lady health workers working all over Pakistan, we estimate that the cost of the national scale-up will be approximately $200,000 for the national program ($100,000 for the cards and pamphlets, and $100,000 for training sessions).

## Conclusion

This study offers firm evidence that providing home-based focused education to mothers regarding the importance of vaccines, through pictorial messages using very simple language, is effective in improving infant immunization rates in low-income and low-literacy populations. Our trial intervention offers novel options to the current vaccine coverage enhancement efforts in low-income countries like Pakistan. Given the low "number needed to treat" to increase immunization completion by 20%, this intervention may prove to be quite cost-effective. The National Lady Health Worker Program is an ideal platform for building a more focused immunization promotion campaign, similar to what we designed, and drive up the demand for infant immunization services in Pakistan. However, the operational and logistical challenges involved in large-scale implementation of such an intervention need further evaluation.

## Abbreviations

EPI: Expanded Program on Immunization; BCG: Bacillus Calmette-Guérin; OPV: Oral Polio Vaccine; DPT: Diphtheria Pertussis Tetanus; CHW: Community health worker.

## Competing interests

The authors declare that they have no competing interests.

## Authors' contributions

AO provided the design and execution of the data analysis, as well as wrote the manuscript. BH contributed to manuscript writing and supervised field operations. ARS supervised manuscript preparation. AA supervised data analysis. AKMZ contributed to the design of the study, data analysis, and manuscript writing. All authors have read and approved the final manuscript.

## Pre-publication history

The pre-publication history for this paper can be accessed here:

http://www.biomedcentral.com/1471-2458/11/239/prepub
